# Effect of prone positioning on inflammatory markers in blood and lungs: a retrospective cohort study in COVID-19-related ARDS

**DOI:** 10.3389/fcimb.2025.1480123

**Published:** 2025-06-30

**Authors:** Chao Hu, Li Peng, Kang Liu, Li Yang, Ying Zhang, Hui Deng, Hui Li, Ming Yan Jiang

**Affiliations:** ^1^ Department of Pulmonary and Critical Medicine, Xiangtan Central Hospital, Xiangtan, Hunan, China; ^2^ Department of Oncology, Xiangtan Central Hospital, Xiangtan, Hunan, China; ^3^ Department of Hematology, Xiangtan Central Hospital, Xiangtan, Hunan, China

**Keywords:** prone position, inflammatory biomarker, bronchoalveolar lavage fluid, COVID-19, lymphocyte subsets

## Abstract

**Introduction:**

Prone positioning is a commonly recommended intervention in clinical practice to enhance oxygenation in COVID-19 patients with ARDS, but its effects on inflammatory markers in the blood and lungs have not been thoroughly investigated.

**Methods:**

This retrospective study examined COVID-19-related ARDS patients admitted to the ICU of a tertiary hospital between January 2020 and November 2023. The analysis focused on measuring cytokines and lymphocyte subsets in both blood and bronchoalveolar lavage fluid (BALF) to evaluate the impact of prone positioning on inflammatory markers in the lungs and systemic circulation.

**Results:**

Of the 86 intubated patients, 44 were included in the study, with 30 undergoing prone positioning. Compared to the supine position, prone positioning was associated with elevated plasma levels of IL-12p70 and IL-4, as well as increased expression of IFN-α and TNF-α in BALF. With increased frequency of prone positioning, oxygenation progressively improved, accompanied by a rise in plasma IL-4 levels. No significant differences in peripheral blood lymphocyte levels were observed between the supine and prone groups.

**Conclusion:**

This study sheds light on the potential mechanisms of prone positioning in both local and systemic circulation, particularly in the context of inflammatory markers. In COVID-19-related ARDS, prone positioning may strengthen the immune response by modulating inflammatory markers in the lungs and bloodstream.

## 1 Introduction

Prone positioning, as a ventilatory treatment strategy, is widely utilized in patients receiving advanced respiratory support (Coppo et al., 2020; J et al., 2022). This approach leverages the anatomical and physiological characteristics of the thoracic wall, where functional stiffening of the ventral chest wall promotes dorsal lung inflation, reducing overall thoracic wall compliance, and enhancing lung homogeneity. This, in turn, facilitates improved ventilation-perfusion (V/Q) matching (Gattinoni et al., 2022). Studies have demonstrated that prone positioning improves oxygenation in 60% to 80% of COVID-19 patients (Camporota et al., 2022; Fossali et al., 2022; J et al., 2022). The correlation between early oxygenation improvement during prone positioning and survival benefits in COVID-19-related ARDS patients substantiates the causal relationship between changes in V/Q matching and clinical outcomes (Scaramuzzo et al., 2021). Consequently, prone positioning has been regarded as a cornerstone in the ventilatory management of critically ill COVID-19 patients (Fossali et al., 2022).

The exploration of the physiological mechanisms underlying prone positioning has been ongoing. Three primary factors—gravity, lung structure and geometry, and the regulation of ventilation and pulmonary vascular tension—are considered crucial influences (Lindahl, 2020). The human respiratory system involves numerous complex and interacting variables. In the prone position, changes occur in the distribution of lung parenchyma within the ribcage, as well as in the positions of the heart and diaphragm (Petersson et al., 2007). The expression of endothelium-derived vasodilators leads to differential vascular regulation between the dorsal and ventral regions of the lungs, counteracting gravity-driven vascular mechanisms (Pelletier et al., 1998). The primary mechanism of prone positioning is understood to improve oxygenation by altering the ventilation-perfusion ratio of alveolar injury exudates, thereby enhancing clinical outcomes. Clinical guidelines recommend initiating prone positioning early in the course of the disease to mitigate localized lung stress and damage (Grasselli et al., 2023). Studie conducted by MUSS et al. has demonstrated that early prone positioning following ICU admission can significantly improve outcomes in COVID-19 patients (Musso et al., 2022). In the early stages of ARDS, patients typically undergo phases of alveolar injury and exudation, which later progress to repair and fibrosis (Thille et al., 2013). Interestingly, some research suggests that even when prone positioning is delayed—initiated after 14 days of intubation—it can still provide clinical benefits (Jackson et al., 2023), suggesting a complex interplay between disease progression and the effectiveness of prone positioning.

Prone ventilation has the potential to not only alleviate lung parenchymal pressure and strain but also to influence inflammatory responses. In ARDS, the elevation of inflammatory cascade markers and the overexpression of cytokines are believed to be indirectly associated with lung stress and injury (Spadaro et al., 2024). Both clinical studies and preclinical animal models have demonstrated that prone positioning can lower plasma IL-6 levels, reducing systemic inflammation, neuronal inflammation, and the neuronal damage associated with ventilator-induced lung injury (VILI), while also enhancing oxygenation (Chan et al., 2007; Yoshida et al., 2021; Sparrow et al., 2022). Despite these findings, most research has concentrated on inflammatory markers in the blood, leaving the effects on pulmonary inflammation less well understood. To address this gap, this study seeks to deepen our understanding of inflammatory dynamics in both the lungs and bloodstream by retrospectively analyzing inflammatory cytokines and lymphocyte levels in the bronchoalveolar lavage fluid (BALF) and blood of a cohort of critically ill COVID-19 pneumonia patients at various stages of prone positioning.

## 2 Methods

### 2.1 Study design and participants

This retrospective study, conducted at Xiangtan Central Hospital, examined adult patients with laboratory-confirmed SARS-CoV-2 infection who required tracheal intubation for ARDS between January 1, 2020, and January 30, 2023. The follow-up period extended until April 30, 2023. To reduce potential confounding effects linked to immunocompromised conditions (Ramirez et al., 2020), the study excluded patients if they had a confirmed human immunodeficiency virus (HIV) infection, an active malignancy within the past year, were receiving immunosuppressive therapy, or lacked bronchoalveolar lavage fluid (BALF) test data. The study was approved by the Ethics Committee of Xiangtan Central Hospital (Approval No.: 2023-03-001), and informed consent for special examinations was obtained from the patients or their families.

### 2.2 Data collection

We collected comprehensive patient demographic data, including age, sex, body mass index, and comorbidities, alongside details of medication treatments. Ventilator settings were meticulously recorded, encompassing mode, positive end-expiratory pressure (PEEP), fraction of inspired oxygen (FiO2), respiratory rate (RR), tidal volume (Vt), and plateau pressure (Pplat). In addition, arterial blood gas measurements—such as partial pressures of oxygen (PaO2) and carbon dioxide (PaCO2)—as well as routine laboratory values, were documented. Blood and BALF samples at baseline were obtained within 72 hours of admission, while post-prone samples were collected within 12 hours following the completion of prone positioning. This data was systematically gathered by a team of physicians with clinical research expertise, utilizing the hospital’s health information system (HIS). We also tracked the application of prone positioning after tracheal intubation and assessed the associated 14-day mortality rate.

### 2.3 Prone positioning

The prone positioning procedure involved a coordinated effort by a team of physicians, nurses, and respiratory therapists, who manually placed the patient in the prone position for a duration of 16 hours before returning them to the supine position. Relevant data, such as the start and end times of prone positioning, mechanical ventilation settings, and vital signs, were obtained from the records maintained by the respiratory therapists and the respiratory rehabilitation team. In the study, “pp0” denotes that no prone positioning was performed, “pp1” indicates that one session was completed, and “pp3” signifies that three sessions were conducted. Oxygen concentration was adjusted according to the patient’s oxygenation status during prone positioning, while respiratory support mode and PEEP levels were maintained unchanged.

### 2.4 Bronchoalveolar lavage fluid

Data for bronchoalveolar lavage fluid (BALF) testing was retrieved from laboratory records in the medical files. Following standard guidelines, BALF collection was initiated in the most affected lung regions (confirmed by a physician with 12 years of experience in respiratory critical care based on chest X-rays or CT scans.). The procedure involved bronchial alveolar lavage using 20 mL aliquots of 0.9% saline at room temperature. Within two hours, at least 60-70% of the lavage fluid was aspirated into a mucus extractor using a syringe and the wedge technique for subsequent analysis. Patients were observed for one hour following the procedure.

### 2.5 Cytokines and peripheral lymphocyte subsets

Flow cytometry (BD FACS Canto II, Becton, Dickinson and Company, BD Biosciences) was employed to analyze a range of cytokines, including IL-6, IL-10, IL-17, TNF-α, IFN-α, IL-1β, IL-2, IL-5, IL-8, IL-12p70, IFN-γ, and IL-4, in both blood and bronchoalveolar lavage fluid (BALF). Blood and BALF supernatant were prepared through a 10-minute centrifugation. Monoclonal antibodies were added to the supernatant and incubated for 120 minutes at room temperature with gentle agitation (400–500 rpm), followed by the addition of SA-PE solution and a subsequent 30-minute incubation under the same conditions, shielded from light. The monoclonal antibodies (Reskell Raisecyte-2L6C, Qingdao, Shandong, China) used for polychromatic cytokine detection included anti-human IL-1β, IL-2, IL-4, IL-5, IL-6, IL-8, IL-10, IL-12p70, IL-17, IFN-γ, TNF-α, and IFN-α. Additionally, the fluorescent monoclonal antibody technique combined with flow cytometry (BD FACS Canto II, Becton, Dickinson and Company, BD Biosciences) was utilized to identify lymphocyte subsets in peripheral blood. 20ulCD3/CD16 + 56/CD45/CD4/CD19/CD8 reagent was added to the bottom of the flow tube, and 50ul EDTA-K2 anticoagulant whole blood was sucked by reverse pipetting technique. The mixture was vortexed for 10s, and then incubated at room temperature in the dark for 15 minutes. The gating strategy is described in **Supplementary Material**.

### 2.6 Statistical analysis

Continuous variables were presented as median with interquartile range (IQR), while categorical variables were shown as frequencies (percentages). The Mann-Whitney rank sum test was employed to compare study groups for non-parametric continuous variables. The Fisher exact or χ2 test was performed for categorical variables accordingly. Friedman repeated measures analysis of variance on ranks or one-way ANOVA for repeated measurements was used to assess differences across time points, based on appropriateness. Using Dunn’s Method, multiple pairwise comparisons were carried out. All statistical tests were two-tailed, with a p-value less than 0.05 considered statistically significant.

## 3 Results

During the period from January 2020 to January 2023, 86 adult patients tested positive for SARS-CoV-2 and required intubation due to acute respiratory distress syndrome (ARDS). After excluding 42 individuals due to immunosuppression, absence of blood test results or bronchoalveolar lavage fluid (BALF) test results, 44 patients were ultimately included in the study (**Supplementary Figure 1**). Of these, 30 patients underwent three sessions of prone positioning, with a 14-day survival rate of 54.5% (20/44). **Table 1** provides an overview of the demographic and clinical characteristics of the patients, categorized by their positioning. Demographic characteristics were similar between supine and prone patients, with the notable difference that the former required slightly lower oxygen concentrations(0.4 vs 0.6, *p* =0.001). Overall, lung-protective ventilation strategies were employed for all intubated patients.

**Table 1:** Intubation COVID-19 patients' characteristics according to position variant.

Variable, median (IQR)	Overall (n = 44)	PRONE (n = 30)	SUPINE (n = 14)	p value
Age (years)	84.72 (77-88)	84.25 (77.5-89)	85.5 (74-87)	0.651
Gender				
Male (%)	36 (81.8)	24 (80)	12 (85.7)	0.651
Comorbidity				
Diabetes (%)	18 (40.9)	12 (40)	6 (42.9)	0.859
hypertension (%)	31 (70.5)	23 (76.7)	8 (57.1)	0.191
Coronary heart disease (%)	24 (54.5)	18 (60)	6 (42.9)	0.293
Chronic lung disease (%)	12 (27.3)	9 (30)	3 (21.4)	0.557
Chronic renal disease (%)	13 (29.5)	10 (33.3)	3 (21.4)	0.425
Respiratory Rate (bpm)	21 (20-23)	21 (20-25)	21 (20-22)	0.512
PaO2/FiO2 (mmHg)	114 (79-164)	95.50 (70.61-143.1)	126 (79-214.3)	0.190
Lactate (mmol/L)	1.8 (1.3-2.5)	1.8 (1.3-2.3)	1.9 (1.0-2.7)	0.728
Glucose (mmol/L	9.0 (6.7-14.4)	8.8 (7.0-13.3)	9.6 (5.9-16.0)	0.623
White blood cell count (*10^9/L)	9.38 (6.94-12.96)	10.33 (8.49-14.60)	9.69 (6.88-13.08)	0.384
Neutrophil (*10^9/L)	9.40 (6.10-12.13)	10.06 (7.67-12.83)	8.69 (5.88-12.13)	0.496
lymphocyte (*10^9/L)	0.6 (0.4-0.9)	0.6 (0.4-0.95)	0.55 (0.38-1.0)	0.849
Monocyte (*10^9/L)	0.43 (0.26-0.73)	0.43 (0.23-0.72)	0.45 (0.28-0.79)	0.641
Alanine aminotransferase (IU/L)	22.0 (13.7-32.8)	22.3 (15.4-34.8)	17.7 (8.5-31.8)	0.290
Albumin (g/L)	34.0 (31.1-37.3)	34.2 (31.2-37.4)	32.4 (30.9-37.4)	0.571
Creatine phosphokinase isoenzyme (IU/L)	16.0 (12.7-21.8)	14.9 (12.4-20.4)	17.7 (14.7-31.3)	0.124
Lactic dehydrogenase (U/L)	365.0 (236.8-520.4)	378.5 (283.0-536.3)	245.0 (193.0-409.0)	0.124
Blood urea nitrogen (mmol/L),	9.8 (5.6-16.9)	8.6 (5.2-14.9)	12.3 (6.5-18.7)	0.427
Creatinine (umol/L)	102.0 (81.3-213.3)	95.0 (80.3-141.8)	129.5 (95.0-666.0)	0.072
C-reaction protein(mg/L)	50.7 (15.6-117.1)	47.8 (11.1-143.5)	53.3 (17.1-88.7)	0.900
Prothrombin time (s)	14.6 (13.3-16.5)	14.1 (13.2-16.0)	14.9 (14.1-26.7)	0.147
Activated partial thromboplastin time (s)	38.9 (31.6-42.6)	38.9 (33.0-43.5)	39.4 (4.4-42.3)	0.821
D-dimer (ng/mL)	1.69 (0.79-16.62)	1.46 (0.74-18.57)	3.36 (1.15-18.98)	0.306
Procalcitonin (ug/L)	0.35 (0.14-6.31)	0.26 (0.14-2.06)	0.91 (0.12-53.82)	0.332
Cstat, median (IQR)	29.17 (20.97-36.81)	28.48 (21.58-36.61)	28.84 (18.71-36.92)	0.936
FiO2, median (IQR)	0.6 (0.4-0.6)	0.6 (0.5-0.71)	0.4 (0.4-0.56)	0.001
PEEP (cmH2O)	10 (9-10)	10 (8-10)	10 (10-10)	0.360
MVe(L/min)	7.9 (6.9-9.7)	8.5 (6.9-10.1)	7.0 (5.45-8.80)	0.109
VTe(mL)	422 (351-487)	416 (353-484.75)	453 (338-516)	0.531

IQR, interquartile range; Cstat, Static lung compliance; FiO2, fraction of inspired oxygen;PEEP, positive end-expiratory pressure; MVe, minute ventilation volume; VTe, tidal volume; PaO2, partial pressures of oxygen.

**Figure 1:**
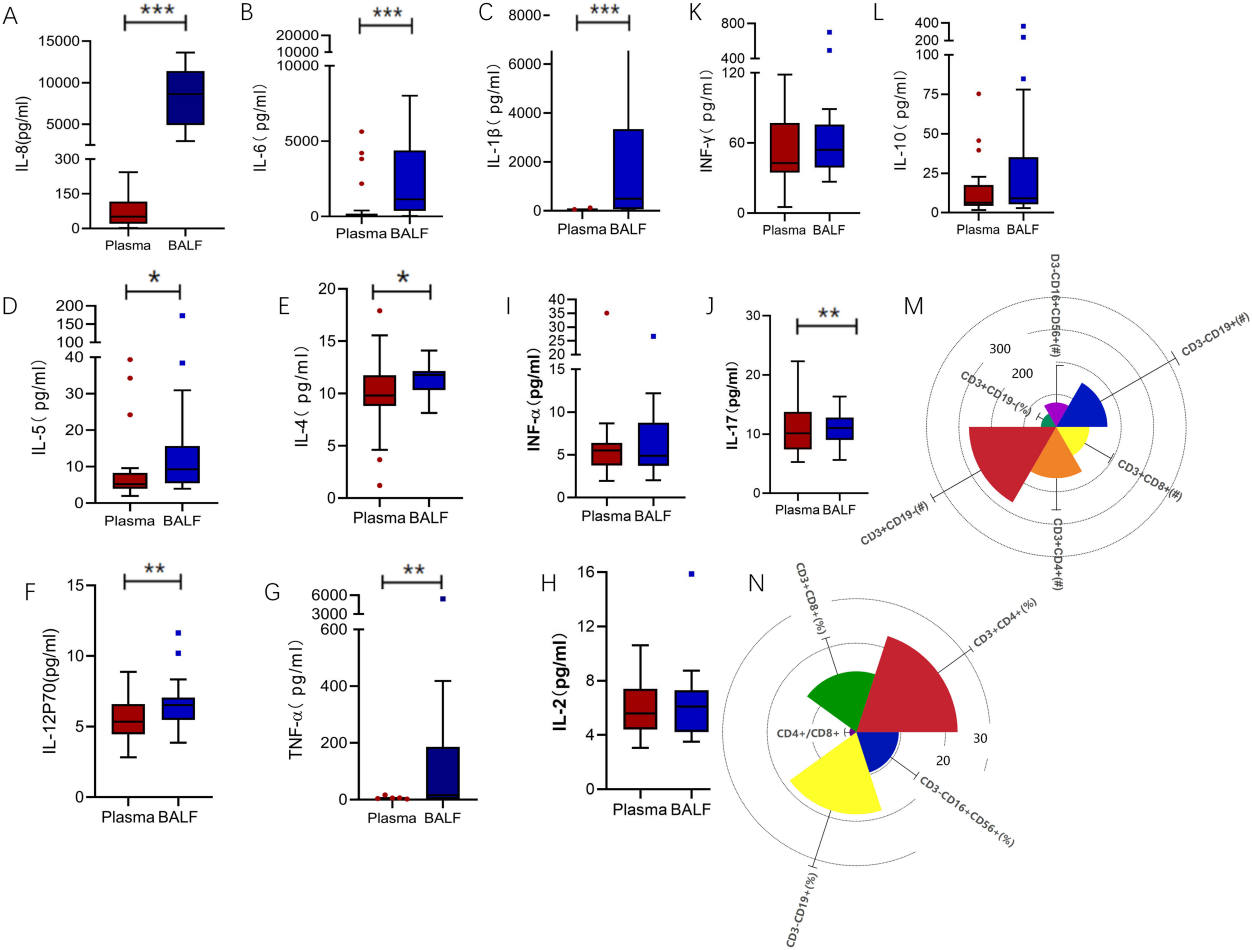
Inflammatory markers biomarkers of BALF and plasma. **(A)** IL-8 levels were significantly higher in BALF compared to plasma (50.7 vs. 8637, *p* < 0.0001). **(B)** IL-6 levels followed a similar trend (42.11 vs. 1139, *p* < 0.0001), as did **(C)** IL-1β (18.08 vs. 496.2, *p* < 0.0001), **(D)** IL-5 (5.19 vs. 9.26, *p* = 0.018), **(E)** IL-4 (9.81 vs. 11.77, *p* = 0.014), **(F)** IL-12P70(5.345 vs. 6.515, *p* = 0.014), and **(G)** TNF-α(2.01 vs. 16.09, *p* = 0.002). In contrast, **(H)** IL-2 (5.61 vs. 6.10, *p* = 0.952), **(I)** IFN-α (5.50 vs. 4.89, *p* = 0.790), **(J)** IL-17(10.17 vs 11.07, *p* = 0.677), **(K)** IFN-γ (42.8 vs. 54.32, *p* = 0.286), and **(L)** IL-10 (6.39 vs. 9.17, *p* = 0.173) showed no significant differences between the compartments. **(M, N)** Radial view of peripheral blood lymphocyte subsets revealed deviations from normal reference ranges. CD3^−^CD19^+^ B cell counts were 196.83PCS /ul (normal: 90–560PCS /ul), CD3^−^CD16^+^CD56^+^ NK cell counts were 94.13PCS /ul (normal: 150–1100PCS /ul), and CD3^+^CD19^−^ T cell counts were 336.16PCS /ul (normal: 955–2860PCS /ul). Subsets of CD3^+^ T cells included CD3^+^CD4^+^ helper T cells at 198.08PCS /ul (normal: 550–1440PCS /ul) and CD3^+^CD8^+^ cytotoxic T cells at 127.80PCS /ul (normal: 320–1250PCS /ul). Proportional analysis showed CD3^+^CD19^−^ T cells at 59.18% (normal: 56–84%), CD3^+^CD4^+^ cells at 34.08% (normal: 27–51%), CD3^+^CD8^+^ cells at 20.53% (normal: 15–44%), CD3^−^CD16^+^CD56^+^ NK cells at 14.29% (normal: 7–40%), and CD3^−^CD19^+^ B cells at 27.71% (normal: 5–18%). The CD4+/CD8+ ratio was elevated at 2.33. **p<0.01, ***p<0.001. BALF, Bronchoalveolar lavage fluid; TNF, Tumor necrosis factor; IL, Interleukin.

Intubated patients demonstrate varying inflammatory responses between the peripheral blood and the lungs, with the peripheral blood reflecting an immunosuppressed state. Inflammatory cytokines, such as IL-8, IL-6, IL-1β, IL-5, IL-4, IL-12p70, and TNF-α, were notably higher in bronchoalveolar lavage fluid (BALF) compared to plasma (**Figures 1A–G**; IL-8: 50.7 vs. 8637, *p* < 0.0001; IL-6: 42.11 vs. 1139, *p* < 0.0001; IL-1β: 18.08 vs. 496.2, *p* < 0.0001; IL-5: 5.19 vs. 9.26, *p* = 0.018; IL-4: 9.81 vs. 11.77, *p* = 0.014; IL-12p70: 5.345 vs. 6.515, *p* = 0.014; TNF-α: 2.01 vs. 16.09, *p* = 0.002). In contrast, levels of cytokines IL-2, INF-α, IL-17, INF-γ and IL-10 remained consistent across both compartments (**Figures 1H–L**; IL-2: 5.61 vs. 6.10, *p* = 0.952; IFN-α: 5.50 vs. 4.89, *p* = 0.790; IL-17: 10.17 vs. 11.07, *p* = 0.677; IFN-γ: 42.8 vs. 54.32, *p* = 0.286; IL-10: 6.39 vs. 9.17, *p* = 0.173). Furthermore, analysis of peripheral blood revealed a reduction in NK cells and T cells compared to standard levels (**Figures 1M, N**). Specifically, CD3^−^CD16^+^CD56^+^ NK cell counts were 94.13PCS/ul (normal range: 150–1100PCS/ul), and CD3^+^CD19^−^ T cell counts were 336.16PCS/ul (normal range: 955–2860PCS/ul).

**Figure 2:**
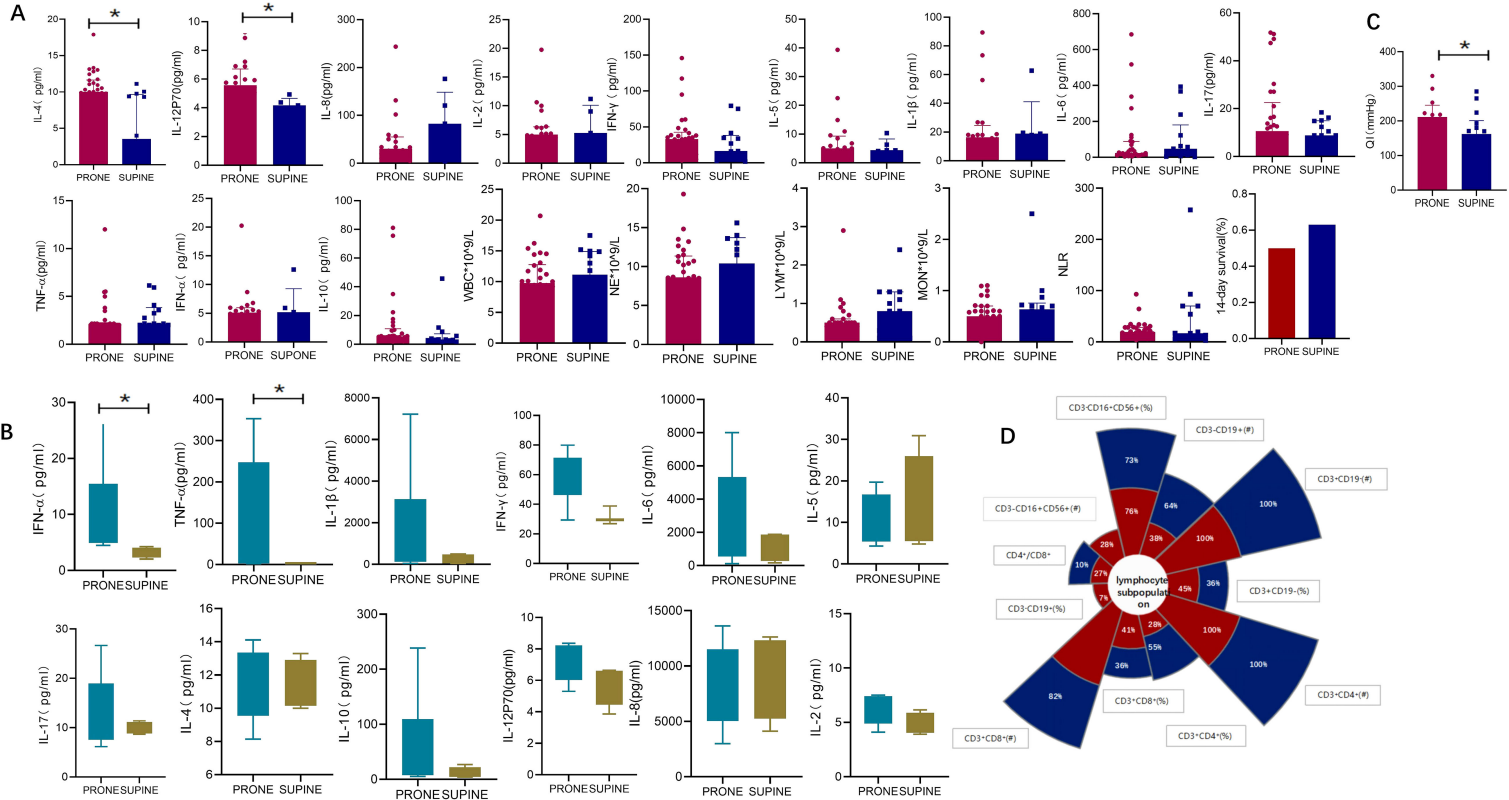
Oxygenation and inflammatory markers in supine and prone positions. **(A)** inflammatory markers in plasma: Plasma levels of IL-12p70 (5.58 vs. 4.17, *p* = 0.015) and IL-4 (10.01 vs. 3.54, *p* = 0.039) were significantly elevated in prone positioning compared to the supine position. No significant differences were observed for other inflammatory markers, including IL-8 (30.10 vs. 82.56, *p* = 0.272), IL-2 (4.99 vs. 5.27, *p* = 0.974), IFN-γ (32.92 vs. 16.38, *p* = 0.300), IL-5 (5.30 vs. 4.39, *p* = 0.922), IL-1β (16.05 vs. 18.90, *p* = 0.497), IL-6 (23.04 vs. 47.48, *p* = 0.788), IL-17 (10.62 vs. 8.82, *p* = 0.297), TNF-α (2.02 vs. 2.17, *p* = 0.118), IFN-α (5.12 vs. 5.20, *p* = 0.912), and IL-10 (5.59 vs. 3.32, *p* = 0.109). Additionally, no significant differences were found in white blood cell (WBC) count (9.78 vs. 11.14, *p* = 0.497), neutrophil count (NE, 8.61 vs. 10.40, *p* = 0.303), lymphocyte count (LYM, 0.50 vs. 0.80, *p* = 0.396), monocyte count (MON, 0.50 vs. 0.63, *p* = 0.252), or the neutrophil-to-lymphocyte ratio (NLR, 19.14 vs. 16.98, *p* = 0.985). The 14-day survival rate showed no significant difference between the groups (0.50 vs. 0.63, *p* = 0.381); **(B)** inflammatory markers in BALF: BALF analysis revealed significantly higher levels of IFN-α (8.75 vs. 3.33, *p* = 0.003) and TNF-α (84.96 vs. 2.01, *p* = 0.036) in prone positioning. Other markers, including IL-1β (1458 vs. 241.3, *p* = 0.148), IL-6 (2395 vs. 1231, *p* = 0.199), IL-5 (6.68 vs. 9.47, *p* = 0.711), IL-17 (11.14 vs. 9.86, *p* = 0.971), IL-4 (11.69 vs. 11.19, *p* = 0.940), IL-10 (11.75 vs. 7.02, *p* = 0.199), IL-12p70 (6.89 vs. 6.37, *p* = 0.106), IL-8 (6085 vs. 10021, *p* = 0.825), IFN-γ (59.80 vs. 29.55, *p* = 0.156), and IL-2 (6.37 vs. 4.76, *p* = 0.113), showed no significant differences between groups. **(C)** Oxygenation(oxygenation index: 218.5 vs. 167, *p* = 0.028). **(D)** The decline rate of peripheral blood lymphocyte subsets. QI, Oxygenation index;BALF, Bronchoalveolar lavage fluid; TNF, Tumor necrosis factor; IL, Interleukin.

**Figure 3:**
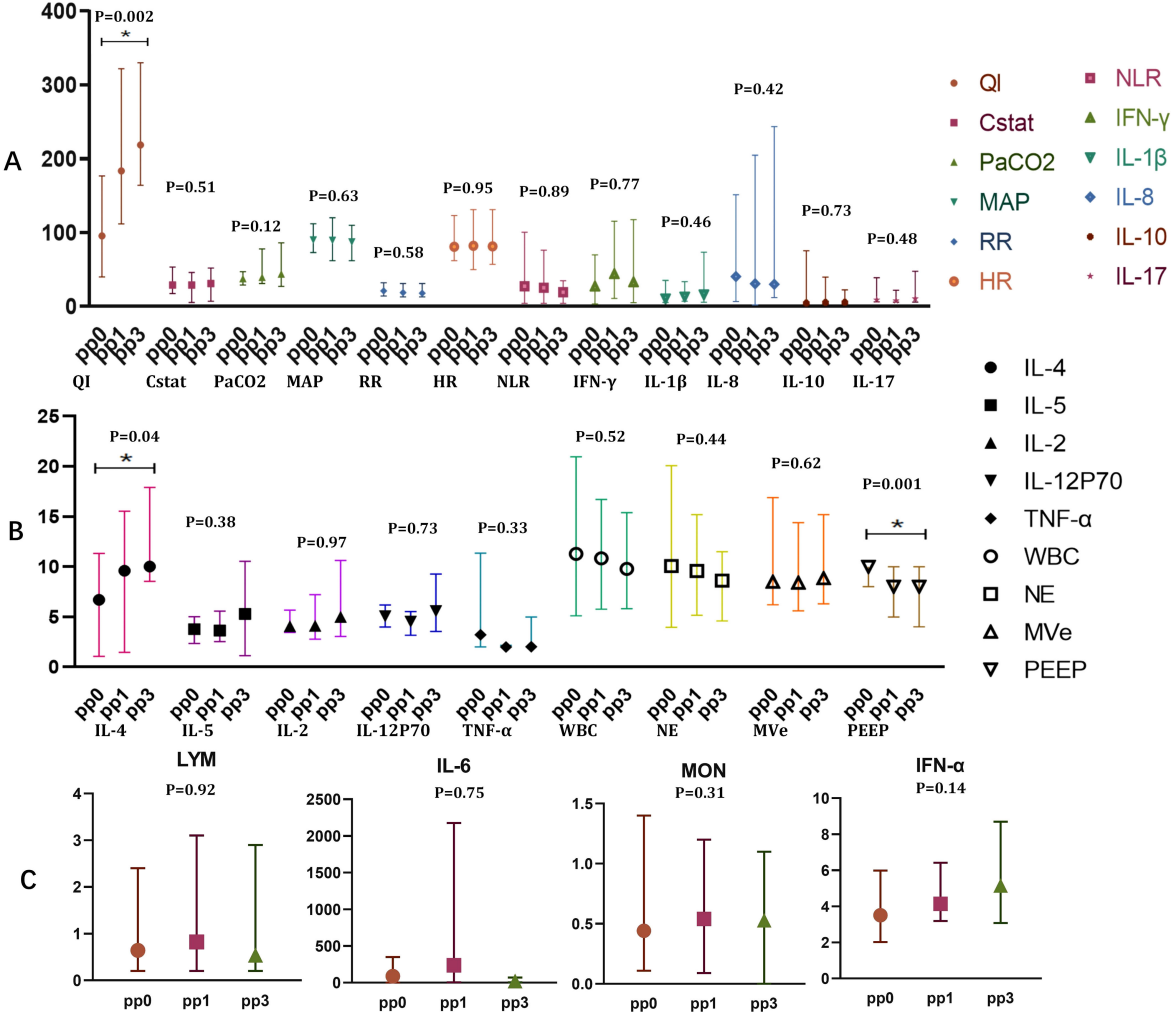
Oxygenation and plasma inflammatory markers of blood in prone position over times. **(A)** QI(95.50 vs. 183.5 vs.218.5), Cstat(28.95 vs. 29.00 vs. 31.11), PaCO_2_(37.5 vs. 39.5 vs. 44.0),MAP(89.97 vs. 89.40 vs. 87.40), RR(21vs. 18.8 vs. 18.03), HR(80.80 vs. 82.22 vs. 81.29), NLR(27.39 vs. 25.25 vs. 19.14), IFN- γ(27.19 vs. 44.00 vs. 32.92), IL-1 β(10.63 vs. 13.16 vs. 16.05), IL-8(40.46 vs. 30.85 vs. 30.1), IL-10(4.45vs5.37vs5.59), IL-17(9.1vs7.79vs10.62); **(B)** IL-4(6.71 vs. 9.59 vs. 10.01), IL-5(3.79 vs. 3.64 vs. 5.30), IL-2(4.06 vs. 4.11 vs. 4.99), IL-12P70(5.05 vs. 4.53 vs. 5.59), TNF-α(3.22 vs. 2.01 vs. 2.02), WBC(10.33 vs. 10.83 vs. 9.78), NE(10.06 vs. 9.91 vs. 8.61), MVe (8.5 vs. 8.4 vs. 8.85), PEEP(10 vs. 8 vs. 8); **(C)** LYM(0.6 vs. 0.82 vs. 0.5), IL-6(87.47 vs. 236.80 vs. 23.04), MON(0.43 vs. 0.54 vs. 0.5),IFN-α(2.92 vs. 4.01 vs.5.12); *p<0.05. PaO_2_, Oxygen partial pressure; FiO_2_, fraction of inspiration O_2_; QI, Oxygenation index;IFN, Interferon; TNF, Tumor necrosis factor; IL, Interleukin.

We next investigated how prone positioning affects oxygenation and cytokine levels. Compared to the supine group, prone positioning significantly increased plasma concentrations of IL-12p70 and IL-4 (**Figure 2A**; IL-12p70: 5.58 vs. 4.17, *p* = 0.0154; IL-4: 10.01 vs. 3.54, *p* = 0.039) and elevated IFN-α,and TNF-α levels in bronchoalveolar lavage fluid (BALF) (**Figure 2B**; IFN-α: 84.96 vs. 2.01, *p* = 0.037; TNF-α: 8.75 vs. 3.33, *p* = 0.003). Other inflammatory cytokines showed no significant differences (**Figure 2**). Following three prone positioning sessions, oxygenation improved (**Figure 2C**; 218.5 vs. 167, *p* = 0.028), although the decline in peripheral blood lymphocyte subsets was similar between the two groups (**Figure 2D**). To further explore the effects of prone positioning duration on inflammatory markers, we analyzed data at three intervals: pp0, pp1, and pp3 (**Figure 3**). Plasma IL-4 levels, a cytokine key to inflammation regulation, significantly increased with extended prone positioning (**Figure 3B**; 6.71vs. 9.59 vs. 10.01, *p* =0.04). Although the changes were not statistically significant, plasma IL-8 levels gradually declined (40.46 vs. 30.85 vs. 30.1; *p* = 0.42), while IL-10 and IFN-α levels rose over time (**Figures 3A, C**; IL-10: 4.45 VS. 5.37 VS. 5.59; *p* = 0.73; IFN-α: 2.92 VS. 4.01 VS. 5.12; *p* = 0.14). Additionally, oxygenation progressively with increased prone positioning sessions (95.5 vs. 183.5 vs. 218.5; *p* = 0.002), while PaCO2 remained stable throughout (**Figure 3A**; 37.5 vs. 39.5 vs. 44.0; *p* = 0.12). Mechanical parameters, such as static lung compliance (Cstat) and PEEP, also showed improvement following three prone positioning sessions (**Figure 3**, Cstat: 28.95 vs. 29.00 vs. 31.11; *p* = 0.51; PEEP: 10 vs. 8 vs. 8; *p* = 0.001).

## 4 Discussion

In this study, we examined the impact of prone positioning on inflammatory markers in both the lungs and blood. Our results show that, compared to the supine position, prone positioning increases IL-4 and IL-12p70 levels in the blood, as well as IFN-α and TNF-α levels in the airways. Additionally, extended prone positioning was linked to shifts in both local and systemic inflammatory markers, alongside improved oxygenation. These findings suggest that prone positioning not only improves lung homogeneity but also modulates inflammatory responses in the lungs and blood, providing valuable insights into the mechanisms underlying this therapeutic approach.

Prone positioning in critically ill COVID-19 patients uncovers distinct immune responses in the airways and blood, characterized by differential shifts in inflammatory markers. In the blood, prone positioning results in elevated IL-4 and IL-12p70 levels, indicating an anti-inflammatory response. In contrast, increased levels of IFN-α and TNF-α in the airways suggest a more pronounced pro-inflammatory reaction. IL-4, a key anti-inflammatory cytokine, is known to significantly inhibit pro-inflammatory cytokines such as TNF-α and IL-1β, enhancing Th2-mediated immune responses and reducing the inflammatory cascade (Renu et al., 2020; Hasanvand, 2022). IL-12p70 has been shown to suppress viral replication and enhance the quality of CD8^+^ T cell responses during viral infections (Costela-Ruiz et al., 2020; Hasanvand, 2022). However, the roles of IFN-α and TNF-α in different tissue contexts remain controversial.

As a type I interferon (IFN-I), IFN-αactivates JAK-STAT signaling, inducing interferon-stimulated genes (ISGs) that orchestrate antiviral immunity (Soltani-Zangbar et al., 2022). Yet, its regulatory effects in COVID-19 are multifaceted. Queiroz et al. reported that severe and long COVID-19 cases exhibit STING and cGAS pathway activation, coinciding with increased IFN-α expression, suggesting a role beyond acute viral clearance and a potential contribution to disease progression (Queiroz et al., 2024). Additionally, other studies have linked elevated serum IFN-α/β levels with increased IL-6 and TNF-α expression but reduced T cell counts (Sun et al., 2023), implying that IFN-α hyperactivation may contribute to immune dysregulation and T cell exhaustion. Our study indicates that prone positioning may elevate IFN-α levels in bronchoalveolar lavage fluid, suggesting a role in local immune modulation. However, given the potential tissue-specific effects of IFN-α, further investigations are required to delineate its dynamic regulation and clarify its role in the immunomodulatory effects of prone positioning. Emerging evidence suggests that type III interferons (IFN-λ) are also elevated in the BALF of severe COVID-19 patients (Broggi et al., 2020). Unlike IFN-I, IFN-λ signals primarily through the IFNLR1 receptor pathway, predominantly acting on epithelial barriers and inducing ISG expression with reduced systemic inflammation (Schuhenn et al., 2022). Due to its localized effects, IFN-λ has been proposed as a potential immunomodulator in COVID-19. However, its precise role in disease progression remains to be fully elucidated. While our study did not assess IFN-λ, future research should investigate whether prone positioning affects IFN-λ expression and its potential role in COVID-19 immune regulation, providing deeper insights into the distinct functions of interferon subtypes in the disease course.

TNF-α, a key pro-inflammatory cytokine, plays a dual role in COVID-19. While it contributes to early antiviral immune defense (Esen et al., 2023), persistent TNF-α signaling may drive disease progression (Popescu et al., 2022). In our study, TNF-α levels remain stable in the blood following prone positioning but increased in BALF, suggesting localized inflammatory regulation without inducing systemic imbalance. Although TNF-α inhibitors have been considered as potential therapeutic agents, evidence suggests that their use may raise the risk of co-infections (such as bacterial and mycobacterial) in COVID-19 patients, potentially worsening the disease (Guo et al., 2022). Therefore, future studies should investigate the spatial and temporal dynamics of TNF-α regulation across different tissue compartments, which may provide valuable insights into its immunomodulatory role in COVID-19. Taken together, these findings imply that prone positioning modulates inflammatory response in COVID-19-related ARDS patients through distinct immune mechanisms, exerting differential effects in the lungs and blood. These variations may reflect the different pathophysiological characteristics of COVID-19 in these tissues (Saris et al., 2021).

Severe COVID-19 patients display a comprehensive immune response marked by T cell exhaustion, excessive cytokine release, and other immune alterations (Yang et al., 2021). In COVID-19-related ARDS, inflammatory marker amplification is more pronounced in the airways than in the bloodstream. In line with previous research (Sparrow et al., 2022), we found significantly elevated levels of IL-6, IL-8, and IL-1β in bronchoalveolar lavage fluid compared to plasma. The activation of lung macrophages in COVID-19 initiates a strong cytokine cascade, and the subsequent release of damage-associated molecules after the acute inflammatory phase contributes to a persistent inflammatory state (Moser et al., 2022). Prone positioning enhances ventilation-perfusion matching by affecting lung stress and compliance (Camporota et al., 2022; Fossali et al., 2022; Gattinoni et al., 2022), which may reduce inflammation and limit injury to the alveolar epithelium and endothelium. Positional changes also influence systemic inflammatory markers. With prolonged prone positioning, plasma IL-4 levels gradually increase, along with improved oxygenation, suggesting that this intervention may alleviate the inflammatory cascade via the IL-4 pathway. In contrast, the rise in cytokines following COVID-19 infection is linked to T cell exhaustion, contributing to disease progression. Apoptosis-related genes such as *IRF1, TP53*, and *CASP3* are upregulated in T cell populations in COVID-19 patients (Zhu et al., 2020).

A reduction in circulating lymphocytes, particularly T cells, is a key feature of the immune response in severe COVID-19 (Zhou et al., 2020), as confirmed in our study. This reduction in immune cells may be driven not only by immune exhaustion but also by the action of cytokines (Tarique et al., 2022). For instance, IFN-α and TNF-α may promote T cell accumulation in lymphoid tissues by facilitating their adhesion to endothelial cells, thereby influencing T cell counts in peripheral blood (Shiow et al., 2006). Furthermore, IL-6 can trigger T cell apoptosis through the Fas-FasL interaction, a mechanism further corroborated by autopsy findings in COVID-19-related ARDS cases (Xu et al., 2020; Xiang et al., 2021). However, our study did not observe significant effects of positional changes on blood lymphocyte subsets, likely due to the limited sample size, emphasizing the need for further investigation in larger cohorts.

Unexpectedly, our study did not observe a significant difference in IL-6 levels between the supine and prone groups, which contrasts with earlier research (Chan et al., 2007). IL-6 plays a key role in the cytokine storm associated with COVID-19, as pro-inflammatory monocyte-derived macrophages contribute to lung injury by secreting high levels of this cytokine (Alon et al., 2021; Yang et al., 2020). A reduction in IL-6 has been closely associated with better outcomes in ARDS patients caused by community-acquired pneumonia (Chan et al., 2007). Although prone positioning improved oxygenation in our study, it did not enhance the 14-day survival rate, potentially explaining the absence of a significant change in IL-6 levels. Another noteworthy finding was the stability of IFN-γ levels in both bronchoalveolar lavage fluid and plasma before and after prone positioning. The role of IFN-γ in viral infections is complex; it is a critical factor in determining the severity of inflammation in Th1-type infections but offers therapeutic benefits in Th2-type infections (De and A, 2001; S et al., 2021; Hu et al., 2022; Yin et al., 2005). The dynamic nature of Th1/Th2 immune responses in COVID-19 is reflected in the fluctuations of IFN-γ levels (Hasanvand, 2022; Gadotti et al. 2020). Viral load is a key determinant of cytokine levels, including IFN-γ, and ultimately influences the likelihood of a cytokine cascades (Hasanvand, 2022; Gadotti et al., 2020). In our study, the difference between plasma IFN-γ levels and those in BALF was not statistically significant, with only a slight increase observed after prone positioning. This may be attributed to the use of small-molecule antiviral drugs that lower viral load, thereby reducing the stimulus for cytokine secretion, though this hypothesis warrants further investigation.

This study has several limitations that warrant consideration. First, as a retrospective analysis, the sample size was limited by the small number of patients who had undergone bronchoalveolar lavage fluid (BALF) testing, which may introduce bias. Second, the absence of continuous monitoring of BALF inflammatory markers over time after prone positioning restricts our ability to assess the dynamic impact of this intervention on local inflammation. Additionally, the study did not evaluate immune cell populations within BALF, limiting insights into the immune cell dynamics. Nevertheless, despite these limitations, the study advances our understanding of the mechanisms underlying prone positioning by comparing its effects on inflammatory markers in both the lungs and blood.

## 5 Conclusion

The study demonstrates that prone positioning enhances oxygenation by improving lung homogeneity through pulmonary physiology and also modulates inflammatory factors both locally in the lungs and systemically. In intubated COVID-19 patients, prone positioning elicits distinct inflammatory profiles in the lungs and blood, promoting pro-inflammatory and antiviral responses in the lungs while supporting anti-inflammatory and antiviral effects in the bloodstream. However, given the limited sample size, the findings should be interpreted with caution, and further studies with larger cohorts are needed to validate these results. Despite this limitation, this research provides valuable insights into the potential mechanisms of prone positioning in both local and systemic circulation by focusing on inflammatory markers in a cohort of intubated COVID-19 patients.

## Data Availability

The original contributions presented in the study are included in the article/**Supplementary Material**. Further inquiries can be directed to the corresponding authors.
