# Supplementary material for: Effect of prone positioning on inflammatory markers in blood and lungs: a retrospective cohort study in COVID-19-related ARDS
Source: Front Cell Infect Microbiol. 2025 Jun 30;15:1480123. doi: 10.3389/fcimb.2025.1480123 (PMC12256543; doi:10.3389/fcimb.2025.1480123)
Supplement: Supplementary file 1 [file DataSheet1.pdf]

1

Director:

Panel: 6 Color TBNK + TruC
Acquired: 1/6/2023 1:15:57 PM
Analyzed: 1/6/2023 1:56:19 PM
TruC Lot ID: 180802
Bead/Pellet: 47150
Status: OK
Operator: BD
Reviewer:
Results: 06012023.csv

Column #1: Column #2: Column #3:

BD FACSCanto II 1 BD FACSCanto v.3.1.5878.21241

CD3/CD16+56/CD45/CD4/CD19/CD8 TruC

Total Events: 10255

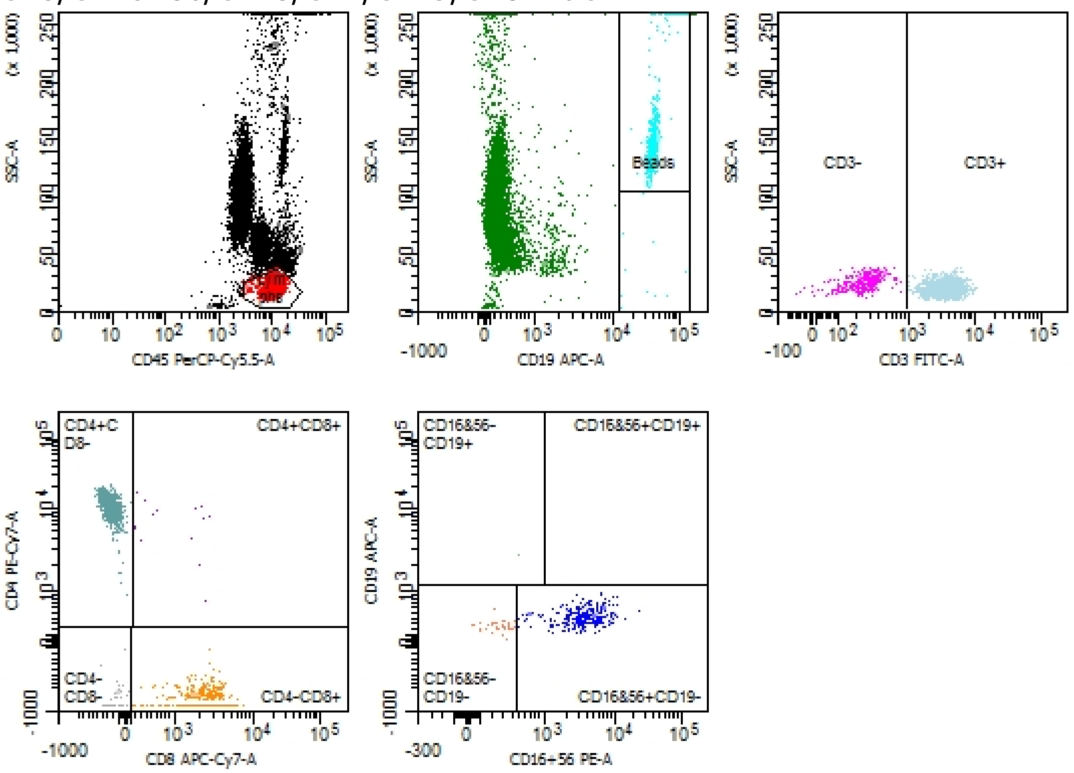

1001.001.fcs

Reagent Lot ID: 181121

Parameter	Percent	Value/AbsCnt
Lymph Events		2254
Bead Events		528
CD3+	85.94	3459.45
CD3+CD8+	28.22	1135.89
CD3+CD4+	56.39	2269.99
CD3+CD4+CD8+	0.67	26.79
CD16+CD56+	12.33	496.50
CD19+	0.04	1.79
CD45+		4025.61
4/8 Ratio		2.00

**1**		
Director:		Panel: 6 Color TBNK + TruC<br>Acquired: 1/6/2023 1:15:57 PM<br>Analyzed: 1/6/2023 1:56:19 PM<br>TruC Lot ID: 180802<br>Bead/Pellet: 47150<br>Status: OK<br>Operator: BD<br>Reviewer:<br>Results: 06012023.csv
Column #1:	Column #2:	Column #3:

BD FACSCanto II 1 BD FACSCanto v.3.1.5878.21241

QC Messages

Manual Gate is in effect.
% T-Sum is: 1.33
Lymphosum is: 98.31
4/8 ratio is: 2.00

Comments
