# Supplementary figures and images for: Effect of prone positioning on inflammatory markers in blood and lungs: a retrospective cohort study in COVID-19-related ARDS
Source: Front Cell Infect Microbiol. 2025 Jun 30;15:1480123. doi: 10.3389/fcimb.2025.1480123 (PMC12256543; doi:10.3389/fcimb.2025.1480123)

FACSCantoll (1)

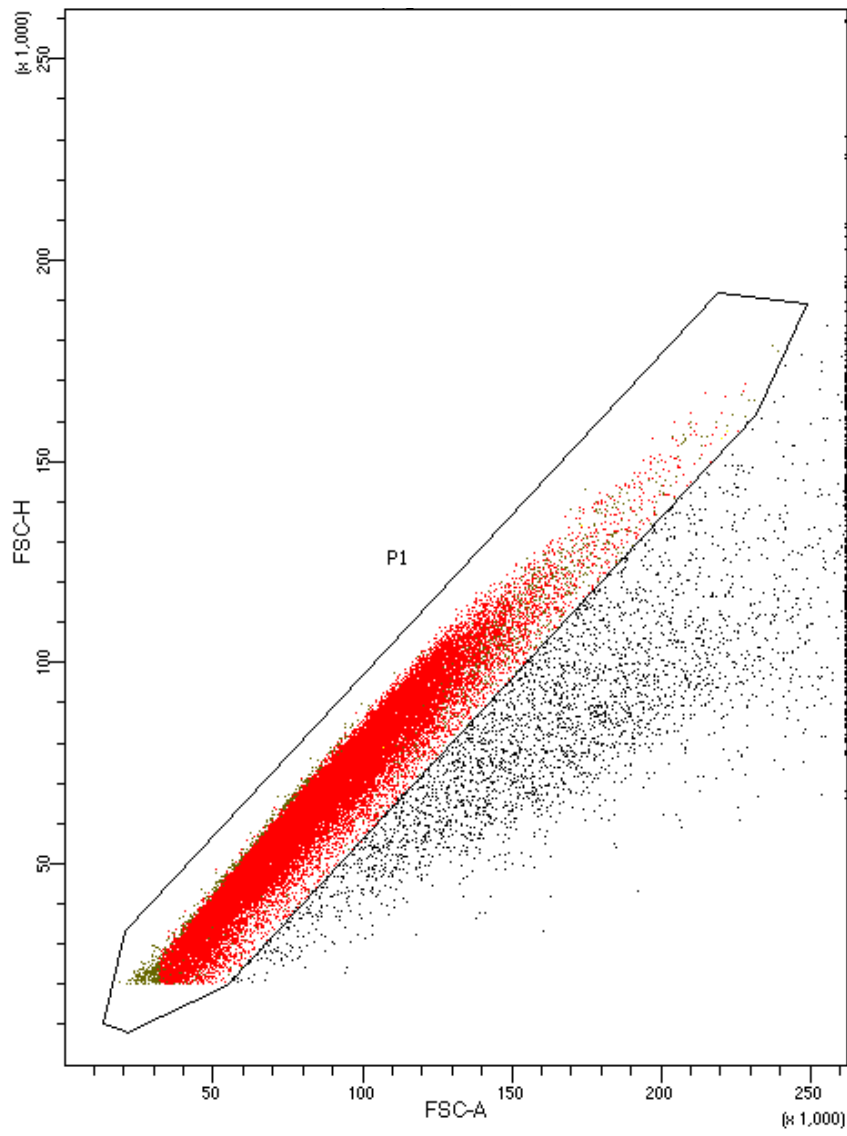

Supplement: Supplementary file 2 [file DataSheet2.pdf]

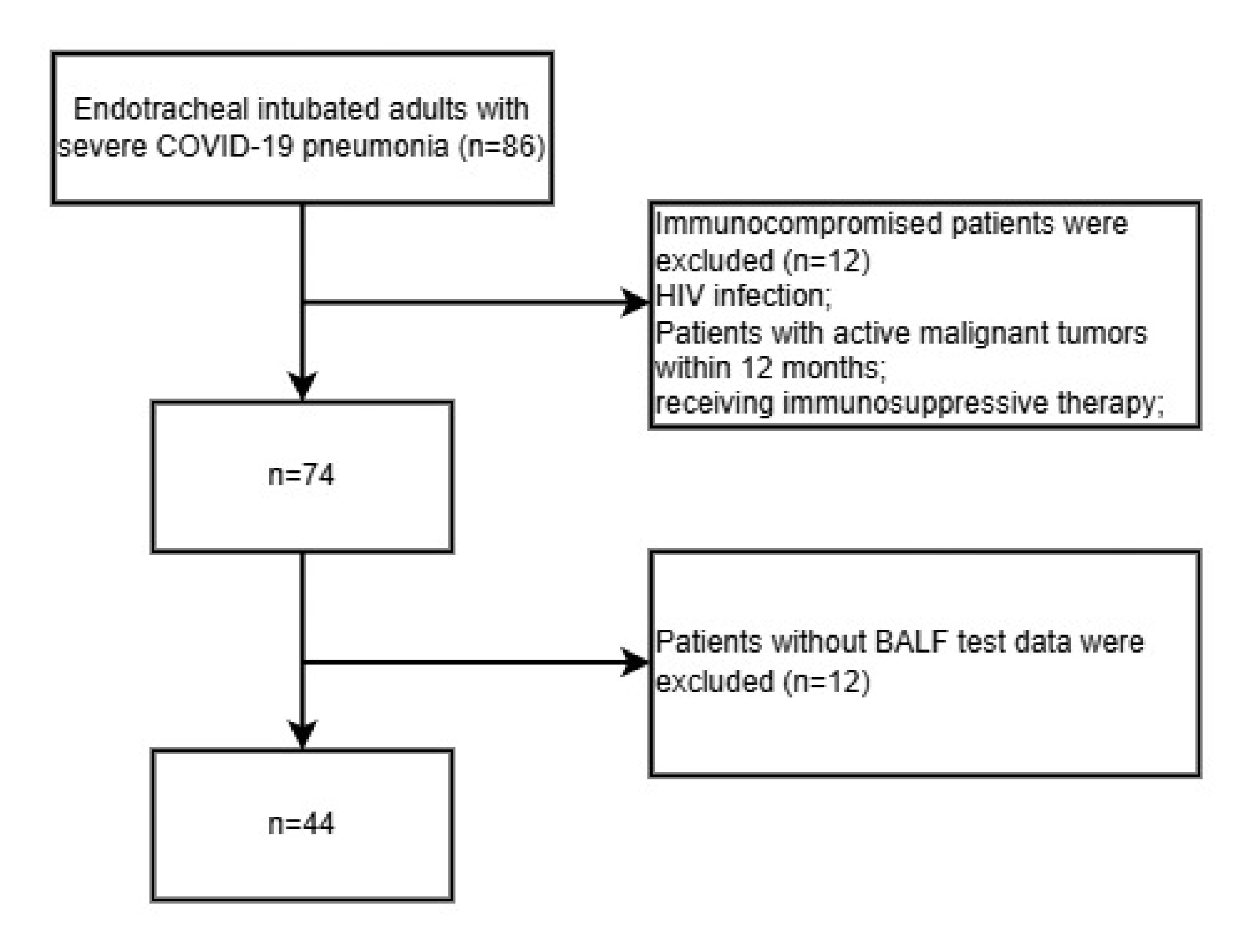

Supplement: Supplementary file 3 [file Image1.png]

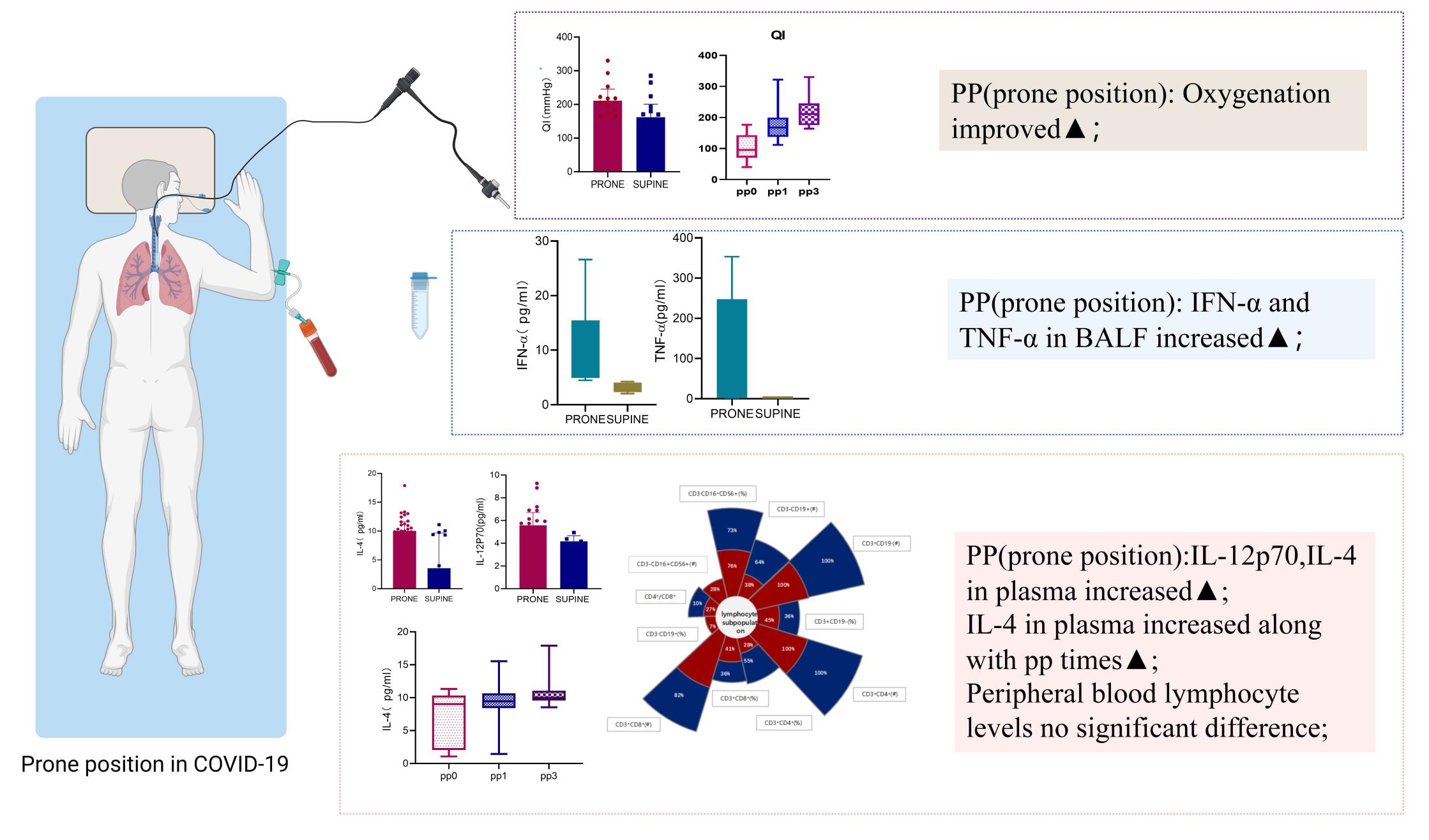

Supplement: Supplementary file 4 [file Image2.jpeg]
